# Feminist approaches to promoting researcher well-being through collective and organizational care

**DOI:** 10.3389/fsoc.2024.1322903

**Published:** 2024-11-06

**Authors:** Catherine Carlson, Sylvia Namakula, Agnes Grace Nabachwa, Anik Gevers, Kelsey Morgan-Babikov, Luciana Giorgio Cosenzo, Melissa Ticozzi, Sophie Namy

**Affiliations:** ^1^University of Alabama, School of Social Work, Tuscaloosa, AL, United States; ^2^Healing and Resilience After Trauma, Kampala, Uganda; ^3^Independent Consultant, Sexual Violence Research Initiative, Pretoria, South Africa; ^4^EverFree, San Juan Capistrano, CA, United States; ^5^School of Social Ecology, University of California, Irvine, Irvine, CA, United States

**Keywords:** vicarious trauma, research methods, ethics, gender-based violence, violence against women, violence against children, collective care

## Abstract

Researchers working in the field of violence against women and children are often tasked with listening to highly distressing personal accounts of violence and subsequent trauma. Without proper attention and mitigation strategies, this exposure can lead to vicarious trauma and related symptoms with significant impact on researchers’ well-being. As women are often leading and carrying out violence research, they also experience a disproportionate burden of risk of vicarious trauma symptoms. This case study highlights seven collective care strategies for research implemented by Healing and Resilience after Trauma (HaRT), a feminist organization dedicated to holistic healing among survivors of human trafficking and gender-based violence, whose team is entirely composed of women. Further, it explores how creating and integrating collective care into research protocols can help prevent vicarious trauma and enhance researchers’ emotional well-being as well as positively influence research quality. Qualitative data from researchers involved in the study on these strategies and how they affected their well-being are included. The piece concludes by discussing potential recommendations for other research teams and organizations seeking to mitigate the risk of vicarious trauma.

## 1 Introduction

Researchers working in the field of violence against women (VAW) and violence against children (VAC) are often required to listen to highly traumatic personal accounts of violence, which can take a considerable emotional toll (Coles et al., 2014). At times, researchers own well-being is affected and they experience symptoms similar to those of direct trauma survivors, a phenomenon called vicarious trauma (Sexual Violence Research Initiative, 2015). This case study highlights seven collective care strategies to promote researcher well-being and reduce vicarious trauma implemented by Healing and Resilience after Trauma (HaRT), a feminist organization dedicated to holistic healing among survivors of human trafficking and gender-based violence. Specifically, the paper will include: (1) An overview of vicarious trauma and researcher well-being, as situated within collective care and feminist research approaches; (2) Context for the case study, including the HaRT research study from which it arose; (3) Description of the collective and organizational care Core Strategies for researcher well-being (found in the Key Elements section); (4) Recommendations for future VAW and VAC researchers to consider when adopting collective and organizational care approaches to researcher well-being.

Vicarious trauma is the result of being exposed to and empathically listening to stories of trauma, suffering, and violence (Pearlman and Saakvitne, 1995). A study conducted by Nen et al. (2011) with professionals experiencing vicarious trauma found numerous symptoms including flashbacks, nightmares, panic attacks, sleep difficulties, and being hyper-vigilant. These symptoms can result in negative cognitive, emotional, behavioral, and interpersonal changes and are associated with poor work performance (Kim et al., 2021). Research with practitioners who have experienced vicarious trauma reveal that they may be less empathic with their clients and more irritable with colleagues (Ravi et al., 2021). While there are fewer studies available on vicarious symptoms among trauma researchers, existing research suggests that they likely experience similar symptoms (Branson and Bixby Radu, 2018; Wallace and County, 2023).

Specific aspects of researching violence may increase the risk of vicarious trauma. Given the shame and stigma around most forms of VAW and VAC, the disclosure of violence during research may be the first time a survivor shares their traumatic experience. In such cases, the researcher may be explicitly told they are the first person to whom the participant has disclosed their experiences, a dynamic which can result in an increased sense of responsibility and emotional distress for the listener (Kim et al., 2021). Additionally, qualitative methods are frequently used in both VAW and VAC research. Qualitative research calls for researchers to be fully immersed through active listening during interviews and the need to use targeted follow up questions, which can increase the risk for vicarious trauma (Branson and Bixby Radu, 2018; Wallace and County, 2023). Further, the iterative process involved in qualitative research can create an emotional connection between the researcher and the participant, another potential risk factor for vicarious trauma (Williamson et al., 2020).

Other aspects that might contribute to vicarious trauma in researchers are related to workload demands, inadequate training, and social support throughout studies (Sexual Violence Research Initiative, 2010). Data collection activities are often structured in a way that maximizes the number of interviews carried out per day, which creates an overload of traumatic content for the researcher to process (Williamson et al., 2020; Ravi et al., 2021). Moreover, researcher trainings and supervision typically focus on the technical aspects of the research and well-being of participants, and often do not adequately discuss vicarious trauma, the identification of related symptoms, or mitigation measures. When research is carried out without adequate social support, it also increases the risk of vicarious trauma, given that social support is critical for processing emotional experiences that can accumulate during the research process (Helpingstine et al., 2021; Ravi et al., 2021). The focus on participants is enhanced by most ethical review boards, which require protections for the well-being of participants, but do not typically require mitigation measures for the protection of researchers.

Researchers’ personal characteristics can also increase the risk of vicarious trauma. For example, researchers working in the fields of VAW and VAC may themselves be survivors of violence (Bloom, 2003; Aroussi, 2020). In such cases, engaging in the research process may risk triggering their own traumatic memories of violence and trauma-related symptoms (Bloom, 2003). Further, persons working in the field of VAC and VAW research are often women, who are at increased risk of post-traumatic stress disorder (PTSD; Olff, 2017). Although there is limited research on women’s increased risk of *vicarious* trauma, it is possible that women’s increased risk of PTSD may carry over to vicarious trauma symptoms, as well.

Given the increased potential of vicarious trauma in VAW and VAC research and its associated harms, a need exists to further explore and utilize strategies for preventing vicarious trauma among researchers in the field of VAW and VAC, particularly collective care strategies implemented by teams and organizations. According to International Women’s Development Agency (2021), “*Collective care involves taking care of ourselves, while intentionally supporting the care of our colleagues, friends, family, and communities. Collective care recognizes the shared responsibility we all have to ensure well-being in our organizations—and it includes self-care. Collective care improves how we relate to each other, decreases feelings of isolation, and increases collective power and solidarity*.” Despite a growing recognition in the fields of VAC and VAW to move beyond a focus on self-care to collective care, few resources exist on *how* to do this, especially in the context of VAW and VAC research.

Collective and organizational care are situated within feminist, healing justice, disability studies, and critical race approaches to activism and programming and align with feminist theory and research methodologies (Page and Woodland, 2023; Piepzna-Samarasinha, 2018; Raising Voices, 2020; Torres, 2020). Feminist research methodology focuses its attention on the presence of power imbalances and their consequences (COFEM 2018), and it does so through the practice of reflexivity and the action of listening to participants’ experiences (Beckman, 2014; Franks, 2022). Additionally, feminist research methodology is concerned with the role that language plays in analyzing and framing social issues and concerns, the ethics of care, and the researcher’s positionality (Beckman, 2014; Franks, 2022; Deutsch, 2004). By recognizing that “the self mediates all knowledge” feminist research strives for connection and reflection rather than detachment, acknowledging that detachment is ultimately impossible (Beckman, 2014, p. 169).

The following community case study aims to advance the feminist research approaches by providing examples of collective and organizational care strategies embedded into a research study with survivors of human trafficking and gender-based violence in Kampala, Uganda.

## 2 Context

This community case study is a collaboration between the Sexual Violence Research Initiative (SVRI), Healing and Resilience after Trauma (HaRT), and the University of Alabama. It evolved from a series led by SVRI that highlighted research, program, and organizational self and collective care practices (Billing et al., 2022). The case study presented herein focuses on lessons learned from a research project carried out by HaRT and the University of Alabama in 2020. The research project itself was a mixed methods evaluation of the ‘Move with HaRT’ program. This case study describes how the research team integrated self and collective care for researchers into the planning, design, and implementation of the research study. The approaches were co-created by the research team, some from the outset of the study and some evolving organically through listening and collective decision making. We explore how these practices helped to prevent vicarious trauma and promote researcher well-being, and share important insights that may be of relevance to researchers, practitioners, and others involved in similar research processes.

HaRT is a feminist organization dedicated to holistic healing among women and girls who have experienced gender-based violence (GBV), including human trafficking. HaRT’s main program, Move with HaRT, is a 12-week, group intervention that uses a variety of contemplative practices (including yoga, mindfulness activities, positive visualization and breath practices), to support collective healing and community among participants (Healing and Resilience after Trauma, 2024). Both the intervention and the research approach is ‘trauma-informed’ (Harris and Fallot, 2001), which we operationalize as maintaining a focus on physical and emotional safety, choice in decision-making, and prioritizing the person over the work (Namy et al., 2022; Namakula et al., 2021).

In 2020, with a small grant from the University of Alabama, HaRT carried out a mixed methods evaluation of the Move with HaRT program in collaboration with Everfree (formerly Willow International), an anti-trafficking NGO that provides residential and community-based services for survivors. Move with HaRT was implemented at two of Everfree’s shelters in Kampala, Uganda with women and girls who had experienced human trafficking. Most participants had also experienced other traumatic events—including physical and sexual violence.

The 2020 evaluation aimed to explore the program feasibility and potential impact on women and girls’ mental health, as well as their physical and social well-being (Carlson et al., forthcoming). Data collection involved six waves of quantitative surveys, as well as two waves of qualitative interviews. The Covid-19 pandemic significantly impacted the research—prompting a shift to virtual data collection methods following the nation-wide lockdown in Uganda (Namakula et al., 2021).

The research team consisted of two researchers based in Uganda, responsible for overall coordination of the research, administering surveys, conducting in-depth-interviews, transcribing and translating data and providing ongoing contributions to data analysis and study refinements. The co-director of HaRT—based in the USA and Spain—developed the research protocol and provided technical assistance remotely throughout the research. Staff onsite at the EverFree shelter also supported the research through mobilizing and coordinating participants and managing crisis referrals. The backdrop for the evaluation research was the onset of the Covid-19 pandemic, which layered on different types of stressors for the all-women team, as well as for the staff and clients at the EverFree shelter.

This case study developed out of interviews (facilitated by an independent consultant of the SVRI who was not part of the original research team) with the four members of the research team: two HaRT researchers (based in Uganda) and two HaRT co-directors (based in the USA and Spain). After interviewing the four research members about the process of conducting the mixed methods evaluation, collective and organizational care practices put into place, and the impact of those practices, the SVRI representative analyzed the qualitative data from those interviews and created an original document describing these practices (Billing et al., 2022). Subsequently, the four members of the original HaRT research team with input from additional co-authors, coalesced and refined these practices into seven key strategies described in this manuscript. The strategies are supported by qualitative data from interviews with the HaRT research team collected from interviews by the original SVRI representative.

## 3 Key Elements

Below we explore seven core strategies implemented during the mixed methods evaluation study, as well as first-hand experiences in how each strategy impacted well-being and the research overall.

### 3.1 Core strategy 1: building a solid foundation

As an organization, HaRT strives to center care as part of its identity and culture—and this included the design and implementation of the 2020 research. HaRT co-directors were aware of power imbalances inherent in any study with leadership based in the Global North. They were intentional about trying to redress potential inequities through co-creating processes and nurturing non-hierarchical relationships (COFEM, 2022).

Creating and sustaining strong relationships of trust across the HaRT team provided an important foundation for self and collective care practices to evolve. This was helped by the fact that all team members had known and worked together for over 5 years. Trust and safety had been built over time in the following ways:

- Open and regular communication—predominantly via phone, Zoom and WhatsApp.
- Promoting non-hierarchical ways of working including establishing practices of collective decision-making among research team members and ensuring researchers had the autonomy to handle decisions and issues as they arose during processes of data collection.
- Valuing and acting upon feedback from all team members.
- Validating the knowledge and expertise of the Ugandan researchers who had insights and perspectives that the HaRT co-directors may not have had.
- Valuing and respecting one another’s full humanity—which meant taking time to understand each other’s lives outside work.

One of the HaRT researchers explained why a sense of trust and safety was an essential precondition for team members to openly share how the work was impacting them, as well as to request additional support:

*“As a researcher I felt I was respected and accepted by all the other members of the team. Because we had trust and respect, I knew no one would think I was weak if I asked for help” HaRT researcher.*

### 3.2 Core strategy 2: comprehensive preparatory training

Researchers participated in training in best practice for trauma-informed research in advance of the data collection process. Parts of the training explored how to ensure researcher well-being and a care-centered approach for research participants during the study. This included explicit psychoeducation on the phenomenon of vicarious trauma, its symptoms, and the importance of our own mental and physical wellness.

In addition, the team discussed difficult scenarios they might face during data collection and explored how they might respond to them. One scenario explored what researchers could do if someone becomes emotional during an interview and cannot continue. The responses they discussed explored how researchers could provide care for the participant, while also ensuring they could nurture their own well-being.

*“The training gave us permission to stop an interview if someone broke down. We discussed how we can offer [the participant] water and let them know it’s okay to stop. In the training it was also clear that we had the freedom as researchers to step away if we needed to and to take a break. In some research projects I’ve been part of, people only care about the data. But we knew we could stop things if we needed to pause” HaRT researcher.*

The training also suggested ideas for preventing scenarios like the one above from occurring in the first place. For example, the researchers understood they had the freedom to pause, stop or interrupt an interview proactively or in discussion with the participant. They explored how to recognize any non-verbal cues or warning signs of distress in the participant—this included stammering and changes in participants’ speech. After discussing how the first few interviews went, the team also made a revision to the interview guide to include breaks to stretch and move in the middle—allowing participants *and researchers* space away from the discussion of distressing experiences. The training also explicitly told researchers that their well-being was important and if they needed to take a break during an interview for their own well-being that it was ok and encouraged.

### 3.3 Core strategy 3: trauma-informed workload management and fair compensation

The HaRT research team was intentional in the way it structured, scheduled, and designed workstreams to minimize the risk of exposure to vicarious trauma, while also ensuring fair compensation. This trauma-informed workload management included:

- Permission to be flexible. As mentioned above, researchers knew they had the ability to stop an interview and take a pause if a participant became distressed.
- Structured breaks. The data collection calendar created space for breaks between interviews and also in the middle of interviews to allow both researchers and participants time to stretch and pause.
- Integrating moments of joy. The interview guide was adapted to ensure researchers could leave participants on a positive note. At the close of the interview, participants were asked to share something that had made them smile in the past week—which had positive benefits for both the researcher and the research participant.
- Ensuring fair compensation. When developing the budget, the team ensured that Ugandan-based researchers conducting the front-line interviews and enduring the emotional burden of listening to trauma stories were fairly compensated for their work—including time for collective care. Part of fair compensation included providing funds for mobile airtime and/or data and transportation to data collection sites.
- Setting workflow limits. The team decided to cap the number of interviews that any one person could do at three per day, in acknowledgment of the length of each interview and the sensitivity of the topics being discussed. Responsibility for interviewing was equally distributed among the two researchers.

### 3.4 Core strategy 4: connecting and supporting during the research process

Researchers connected and supported each other as an essential strategy for reducing the possibility of vicarious trauma. The HaRT evaluation had a systematic two-tier approach to connecting and supporting one another. The two researchers working on-site checked in with each other after every interview. Interviews were lengthy (up to 1.5 h) and could be emotionally intense. There were also remote connect and support sessions with the entire HaRT team at the end of every day of data collection. The connect and support process aimed to (a) provide emotional support for the researcher; (b) assess for potential vicarious trauma; and (c) determine if any changes needed to be made in the research protocol or schedule to better support the interviewers. Some of the questions that were used in these sessions are included in Table 1. Researchers were reminded to not share any identifying information or details that could break participant confidentiality. Thus, questions focus on researchers’ own feelings, needs, and well-being.

**Table 1:** Connecting and supporting questions.

1. How are you feeling after today’s interview/s? (Follow up if needed: How are you feeling emotionally? What about physically?)
2. Without disclosing any specific story or participant, are there any interviews or stories that you are unable to stop thinking about?
3. What have you been able to do to help yourself manage the difficulty of conducting interviews?
4. What self-care can you do for yourself before the next interviews?
5. Overall, how has the research project been affecting you emotionally/mentally/physically?
6. Is there anything that we can change in the research plans to help you better handle the stress of the research?
7. Do you feel you need a break from conducting interviews?

The two-tier approach to connecting and supporting was found by the researchers to be very important for their own well-being.

*“The connecting and supporting was very useful. I didn’t have to wait until the end of the day as I could speak openly to [my colleague] after each interview. And then, at the end of the day, I knew I would also have the chance to talk to the rest of the team… Having this space made me feel whole again. I knew that what I had heard that day wouldn’t affect me as much” HaRT researcher.*

The facilitators of the connect and support sessions (the HaRT co-directors) also reiterated positive, trauma-informed messages to the researchers—including appreciating and validating their work and reminding them of the ways in which they were having a positive impact through this research. They strived to normalize any emotions that researchers might be experiencing because of the data collection process, and reassured researchers they were part of a team and did not have to handle difficult emotions alone. The researchers particularly appreciated the self-talk strategies they learned during connect and support sessions:

*“The self-talk was very helpful. When we were feeling low about something we had heard, we reminded ourselves that we did not contribute to their [the survivor’s] distress. When we felt bad because we had not been able to do a lot to help the person, we reminded ourselves that we had listened. While we had not been able to provide them with counseling ourselves, we had referred them to a counselor. We told ourselves, ‘I’ve done the best for them that I could do.” HaRT researcher.*

The implementation of this strategy, along with many of the others, required all research members dedicate additional time for discussion and connection—which was not without challenges and sacrifies in balancing other work and life responsibilities. Yet, this connection was found to be valuable and, with integration of supports from Core Strategy 3 (trauma-informed workload management and fair compensation), was worth the time investment.

### 3.5 Core strategy 5: building community and solidarity outside the research process

Risk factors for vicarious trauma were part of the very nature of the research study: the work involved listening to survivors describe their traumatic experiences, overall perceptions of their physical and mental health, and reflections on any recent changes. One of the questions in the survey from the Life Event Checklist-5 (part of a standardized trauma events checklist) asked: ‘What is the worst event that has ever happened to you?’

*“The hardest parts of the research were when young women and girls shared their stories about the worst event that had happened to them. Sometimes, these were things they had never disclosed to anyone before. I was the first person in the world to hear them… you have to listen and be supportive” HaRT researcher.*

As previously mentioned, a lack of social support is a risk factor for vicarious trauma (Helpingstine et al., 2021; Ravi et al., 2021). Nurturing a sense of community and collective care can be an important tool for counteracting research-related vicarious trauma. Within the HaRT research team, well-being was seen as a collective responsibility—for example, when describing their job responsibilities during interviews for this case study, HaRT team members recounted the responsibility they had to extend emotional support to other members of the team. Collective care also took the form of checking in with each other about life outside of work, including asking questions about each other’s family, health or any other issues that were arising at the time. Further, the team ensured that researchers working in Uganda worked together (as a pair) wherever possible—which gave them access to a peer supporter for informal check-ins and support should anything unexpected arise. Peer support can be a useful way to prevent and mitigate research-related vicarious trauma (Hummel and El Kurd, 2021). Without violating participant confidentiality, researchers found the presence of another trusted colleague onsite with whom to visit with (even if about informal day-to-day conversation) helped with relaxation and emotional release in between interviews.

Regular communication between team members facilitated community building and collective care. Because the HaRT team was geographically dispersed, WhatsApp groups were used for informal check-ins, connecting team members in different countries, wishing good luck for the upcoming day’s work, as well as a way of sharing short meditative grounding practices for everyone to use if helpful.

*“Sometimes we also did these short relaxation exercises that are part of the Move with HaRT program ourselves. We shared them on WhatsApp, and they were relaxing! By the time you finish, you feel you have forgotten all the stress. It helps you to put aside issues from the interviews, and focus on your muscles, focus on your arms and legs … on the now of the present moment rather than worrying” HaRT researcher.*

Further, the team was experiencing a unique type of collective stress due to the onset of the COVID-19 pandemic at the beginning of the research. Again, the team centered a collaborative, rather than an individually-centered approach, to promote collective well-being.

For example, during the onset of the pandemic, the team had honest group conversations about whether each person felt ready to continue with the research and to freely discuss whether they had enough stability within themselves to hold space for research participants, given the difficult operating environment.

### 3.6 Core strategy 6: professional psychological support for research team

Another strategy integrated into the research protocol to promote well-being was integrating external psychological support. Researchers were asked if they would want to schedule a session with an external, professional psychologist. This support was optional, and the researchers were invited to have either one individual or two group sessions. The researchers opted to have two group sessions. As emphasized throughout the study, researchers were reminded to maintain participant confidentiality and avoid sharing identifying stories or information. Instead, the purpose of the psychological support was intended to focus on their own emotions and well-being. For both researchers, this was the first time they had personally engaged with a psychological professional and were initially hesitant. However, the option was carefully introduced to explain the purpose, avoid stigma or misunderstanding, and ensure that they understood this was completely voluntary. Both researchers reported that spending time with an external professional enhanced their well-being and their understanding of the importance of researcher care throughout the research process.

*“It was really great to have access to a counselor—she spoke about some of the things we discussed in our connect and share sessions, but in a different way, with an independent eye. She had a different perspective on what we were experiencing that I found very useful… I also found it useful participating in this with my colleague—because I learned so much from her insights, too” HaRT researcher.*

The researchers further elaborated on how this additional support had been invaluable to them as they dealt with the impacts of the Covid-19 pandemic:

*“The external psychologist helped us to balance the research project, our lives and Covid-19. I would definitely recommend this approach to other research teams… I really felt I was working with people who cared about my well-being” HaRT researcher.*

While the researchers found these two sessions be enough, future studies may need to consider reserving resources to support longer-term counselling support for researchers experiencing vicarious trauma.

### 3.7 Core strategy 7: referral pathways for research participants

Researchers may be exposed to emotional distress when, during the course of their work, they recognize that research participants require services or additional support and yet do not have the information on hand or the agency to be able to provide it or make necessary referrals (Sexual Violence Research Initiative, 2010).

A care-centered approach within the HaRT research study involved making the time to ensure referral pathways were in place before the data collection began. This meant that should unforeseen issues arise during the data collection process, the research team would be able to signpost participants to services or refer them for additional support. The study had two types of referrals: voluntary and mandatory. Mandatory referrals occurred with any disclosures of suicide or child maltreatment and was explained to the participant during the informed consent process. Voluntary referrals for additional support with any other issue that arose (non-suicidal emotional distress, intimate partner violence, health concerns, etc.) were offered to everyone at the end of the interviews. Fortunately, the team had access to trained staff at the EverFree shelter who were able to provide immediate case management and follow-up. Time and attention were given to ensure the mandatory and non-mandatory referral criteria and processes were clear (both to the EverFree and HaRT teams), with the acknowledgment that should a referral pathway not be able to meet the needs of the research participant, this could be an additional emotional burden for the researchers to carry.

*“Not all of the risks can be anticipated during a research study like this. For instance, at the very start of the research, we encountered suicidal ideation among several participants, which triggered a mandatory referral process. We had not anticipated the extent this would be needed, and we worked with counselors at [EverFree] to make sure they felt confident to handle risk-to-suicide cases. This involved organizing a training for [EverFree] staff in safety planning for suicide prevention” HaRT co-director.*

Due to the onset of the COVID-19 pandemic, researchers faced additional distress when they observed the extent to which the pandemic was impacting the research participants. During one of Uganda’s lockdowns, some research participants opted to leave the EverFree shelters to return to their home communities. These participants decided to leave the shelter for several reasons, such as wanting to be close to family members for an unforeseeable amount of time, caring for older parents or children, fear of being in the city for a pandemic and feeling safer in the village. With the participants no longer at the shelters, the research team held an immense concern for these women and girls—especially when it became known that some of them were struggling to meet their basic needs outside of the shelter. Together, HaRT and EverFree applied for (and received) an Urgent Action Fund Africa rapid response grant which allowed them to distribute emergency cash payments to those who were struggling to feed and house themselves. This action was not only helpful to the research participants who had transitioned to their home communities, but it relieved an emotional burden on the researchers.

### 3.8 The impact of HaRT’s approach on the team

HaRT’s approach to care and well-being had profound impacts on members of the research team. During the interviews, they spoke at length about how they benefited from the supportive network and practices of collective care that had been embedded in their way of working.

*“At the time of the research, I was a new mother. I had just had a baby. At the same time, we were all handling Covid-19, which added more stress. If I hadn’t had the care offered to me [by the HaRT team], I don’t think I could have continued with the work” HaRT researcher.*

Team members also shared how the approach to care and researcher well-being had a positive impact on the quality of the research:

*“Because the researchers knew they were supported to deal with any of the difficult aspects of the work, they were better able to support the research participants themselves. They were able to bear witness and hold the space. This made them better researchers” HaRT co-director.*

This sentiment was also expressed by a HaRT researcher. The sum of these strategies in creating a nurturing team environment, where the well-being of everyone was prioritized, led her to be able to be a better listener to participants.

*“[In this research study] we really focused on how to collect the data… Usually, in so many research projects you’re made to feel that the most important thing is the data. But I really cared about how the participants were responding and how they were coping with the process. This made me a much better listener—and the research participants noticed that. They felt they were being heard. If a person doesn’t feel like they’re really being listened to, as if they are being bombarded with research questions, they won’t respond well. The data we were able to gather was so much stronger as a result” HaRT researcher.*

One researcher described how she was able to care much more about the quality of the data she was collecting because of the support she was getting for her well-being throughout the research:

*“Researchers who are stressed out all the time aren’t able to care so much about the quality of the data they are collecting. They just know they need to finish the work. This study was different” HaRT researcher.*

## 4 Discussion

HaRT’s approach recognized that individualized self-care strategies may not always be enough to ensure researcher well-being, and that collective care practices play an important role in reducing the risk of vicarious trauma and creating a supportive work environment. The following considerations may be useful to other organizations, research teams, and ethics review committees in developing and reviewing collective care strategies as an essential component of all VAW and VAC research studies:

- Center equal and authentic relationships throughout the work. Although the HaRT research team had worked together for some years, their experience is a reminder of the importance of investing time in building relationships and rapport within research teams. The process of getting to know, trust, and value each other as individuals, instead of solely viewing one another as ‘instruments’ to get the work done, is critical to collective care.
- Adapt research timelines and budgets to center care. The successful implementation of the above strategies required additional time and resources. Although this research project was carried out on a small budget, funding priorities were made to account for fewer interviews per day, daily check-ins, and providing external psychological care sessions. Advocacy is needed with donors to understand that careful, safe, and effective violence research may require lengthier timelines and larger budgets with dedicated funds for self and collective care.
- Include safety and ethics protocols for researchers, as well as participants, in ethical review board protocols. Ethical review boards provide oversight to protect study participants. Yet the importance of vicarious trauma and researcher well-being does not get the same level of attention. Ethical review processes could ask questions about potential risks to researchers and strategies to mitigate those risks.
- Conduct further research on how to prevent vicarious trauma and promote researcher well-being within violence research. Although the HaRT team developed several effective strategies during the evaluation, other approaches may also be useful and needed in different contexts. In general, there is a lack of research on vicarious trauma in the field of violence research—a gap which itself deserves more learning. Further, the effectiveness of the strategies presented in this case study or those used by other groups deserve additional investigation.
- Establish close collaborations between research and program teams. Given their role in providing direct services, many program teams may be more equipped and prepared to address vicarious trauma among staff and can infuse such learnings into research protocols. Close coordination around referrals is also critical to ensure a transparent and reliable approach for crisis referrals when needed, as demonstrated in the HaRT case study.
- Hold collective care front and center as situations change and unexpected needs arise. Research teams must also remain open to unexpected needs that arise throughout the research process, and make real-time adjustments to prioritize the well-being of both participants and researchers.

This community case study highlights how collective care strategies can play an important role in preventing vicarious trauma, creating positive experiences across research teams, and contributing to higher quality research. Such recommendations are likely most applicable to similar studies, for example those working in teams (as opposed to individual dissertation or thesis work). We also acknowledge that these learnings came from a small project with a previously established, all woman research team working in Uganda. Other studies on violence with different sample sizes, focus, team members, culture/s, and/or contexts will likely need to consider different or additional strategies than those described in this case study. Adapting or developing new collective care strategies for different studies and contexts can be best achieved by investing time in open listening and shared decision-making from all members of a research team.

The case study itself also has limitations. For example, it originated from interviews with members of the core research team and did not include interviews of participants themselves or partner organizations. Still, we believe this case study is a timely reminder of the importance of investing in feminist collective care practices within research on VAW and VAC. Adoption of such an approach from the outset of a study can not only promote researcher well-being, but enhance overall research ethics and quality.

## Data Availability

The original contributions presented in the study are included in the article/supplementary material, further inquiries can be directed to the corresponding author.
